# Strain wave pathway to semiconductor-to-metal transition revealed by time-resolved X-ray powder diffraction

**DOI:** 10.1038/s41467-021-21316-y

**Published:** 2021-02-23

**Authors:** C. Mariette, M. Lorenc, H. Cailleau, E. Collet, L. Guérin, A. Volte, E. Trzop, R. Bertoni, X. Dong, B. Lépine, O. Hernandez, E. Janod, L. Cario, V. Ta Phuoc, S. Ohkoshi, H. Tokoro, L. Patthey, A. Babic, I. Usov, D. Ozerov, L. Sala, S. Ebner, P. Böhler, A. Keller, A. Oggenfuss, T. Zmofing, S. Redford, S. Vetter, R. Follath, P. Juranic, A. Schreiber, P. Beaud, V. Esposito, Y. Deng, G. Ingold, M. Chergui, G. F. Mancini, R. Mankowsky, C. Svetina, S. Zerdane, A. Mozzanica, A. Bosak, M. Wulff, M. Levantino, H. Lemke, M. Cammarata

**Affiliations:** 1 Univ Rennes, CNRS, IPR (Institut de Physique de Rennes)—UMR 6251, Rennes, France; 2 Univ Rennes, CNRS, ISCR (Institut des Sciences Chimiques de Rennes)—UMR 6226, Rennes, France; 3 Institut des Matériaux Jean Rouxel (IMN), Université de Nantes, CNRS, Nantes, France; 4 GREMAN—UMR 7347 CNRS, Université de Tours, Tours, France; 5 Department of Chemistry, School of Science, The University of Tokyo, Bunkyo-ku, Tokyo Japan; 6 Department of Materials Science, Faculty of Pure and Applied Sciences, University of Tsukuba, Tsukuba, Ibaraki Japan; 7 SwissFEL, Paul Scherrer Institut, Villigen PSI, Switzerland; 8 Laboratory of Ultrafast Spectroscopy, Lausanne Center for Ultrafast Science (LACUS), École Polytechnique Fédérale de Lausanne, Lausanne, Switzerland; 9 European Synchrotron Radiation Facility, Grenoble, France; 10 Present Address: Institute for Materials and Energy Science, Stanford University and SLAC National Accelerator Laboratory, Menlo Park, CA USA; 11 Present Address: European Synchrotron Radiation Facility, Grenoble, France

**Keywords:** Phase transitions and critical phenomena, Electronic properties and materials

## Abstract

One of the main challenges in ultrafast material science is to trigger phase transitions with short pulses of light. Here we show how strain waves, launched by electronic and structural precursor phenomena, determine a coherent macroscopic transformation pathway for the semiconducting-to-metal transition in bistable Ti_3_O_5_ nanocrystals. Employing femtosecond powder X-ray diffraction, we measure the lattice deformation in the phase transition as a function of time. We monitor the early intra-cell distortion around the light absorbing metal dimer and the long range deformations governed by acoustic waves propagating from the laser-exposed Ti_3_O_5_ surface. We developed a simplified elastic model demonstrating that picosecond switching in nanocrystals happens concomitantly with the propagating acoustic wavefront, several decades faster than thermal processes governed by heat diffusion.

## Abstract (web summary)

Ultrafast control of materials draws interest. Here, the authors extend X-ray powder diffraction to the femtosecond timescale to follow the photo-induced semiconductor to metal transition in titanium pentaoxide, observing a phase front that moves at the speed of sound and proposing a little explored mechanism.

## Introduction

New opportunities have emerged for transforming materials with femtosecond (fs) laser pulses^1–5^. New breakthroughs are anticipated such as attaining macroscopic transformations by driving the material on a deterministic pathway from one phase to another, very fast and efficiently. During these transformations different degrees of freedom couple sequentially and give rise to multiscale dynamics in space and time. Some of these degrees of freedom result in structural reorganizations that are crucial for stabilizing a photoexcited electronic state. Many studies have focused on optical coherent phonon oscillations around the new atomic positions^6,7^. However, these intracell atomic displacements preserve the crystal shape and volume. The stabilization of a new macroscopic structural order requires long-range crystal deformations that involve propagating acoustic waves. In a recent study of spin-crossover molecular crystals we showed that cooperative elastic interactions help to form a macroscopically robust switched state^8^. In another study of a photo-induced insulator to metal transition, it was proposed that local expansion of the lattice drives the propagation of the metallic region^9^. In order to examine the role of the lattice strain in photo-induced phase transition^10^, we investigate the semiconductor-to-metal (S-M) phase transition in Ti_3_O_5_ nanocrystals that exhibit even greater volume change, and a high resilience to intense laser pulses. In the aforementioned studies^8,9^, transient optical spectroscopy was used to follow the photo-induced changes. Unfortunately optical observables are hard to relate to structural changes, like lattice deformations. In this contribution we use X-ray pulses from an X-ray Free Electron Laser (XFEL) and synchrotrons to perform time-resolved powder diffraction, allowing a quantitative refinement of the structures on fs to μs time scales. The ultrafast strain dynamics have been further rationalized by adopting a well-known phenomenological elastic model^11,12^ and accounting for phase transformation. The highly sensitive diffraction patterns revealed the structural changes acting as precursor stress and resulting in the macroscopic phase switching that moves as a strain wave at the speed of sound, hereafter referred to as the acoustic front.

**Fig. 1:**
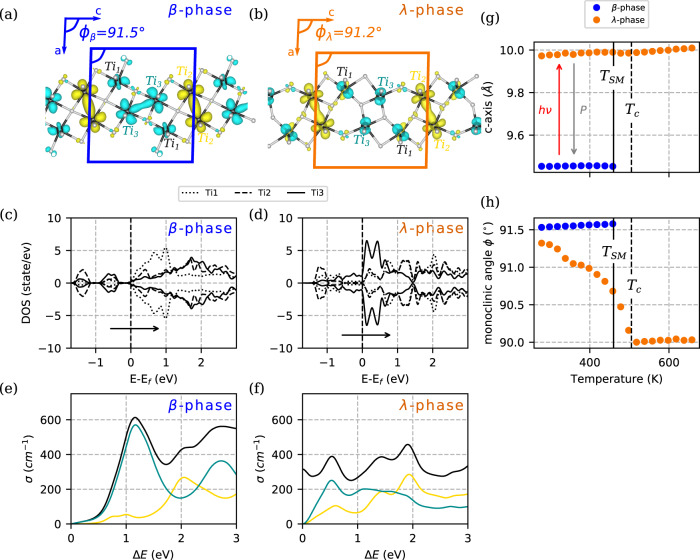
Structural and optical changes upon phase transition in Ti_3_O_5_ nanocrystals at thermal equilibrium. **a**, **b** Atomic structure of the β- and λ- phases, respectively, with the calculated charge density for the two bands centered at 1.1 eV (yellow) and 0.3 eV (cyan) below the Fermi level, strongly localized around *Ti*_*2*_*-Ti*_*2*_ and *Ti*_*3*_*-Ti*_*3*_ dimers; ϕ_β_ and ϕ_λ_ are the monoclinic angles of respectively β- and λ- unit cell. **c**, **d** Calculated density of state projected on Ti_1_ (dotted lines), Ti_2_ (dashed lines), and Ti_3_ (solid lines) in the β- and λ- phases, respectively. The black arrow is the energy transfer corresponding to the 1.55 eV pump photons. **e**, **f** Mean calculated optical conductivity (σ) for β- and λ- phases, respectively (black line). Yellow (cyan) curve is the contribution from the band at −1.1 eV (−0.3 eV). Diagonal contributions to the conductivity are shown in Fig. S1. **g**, **h** X-ray powder diffraction data on Ti_3_O_5_ nanocrystals: **g** change in c-parameter of the monoclinic unit cell with a jump showing the first order phase transition at *T*_SM_ = 460 K between the β- and λ-phases; **h** Temperature dependence of the monoclinic angle *ϕ* with the first order phase transition at *T*_SM_ = 460 K (between the β- and λ-phases which co-exist in these nanocrystals below *T*_SM_), and a continuous locking at 90° characterizing the symmetry change towards the *Cmcm* high temperature α-phase. The critical temperature *T*_c_ = 500 K for this second order phase transition is shown with a dotted vertical line.

The trititanium pentoxide (Ti_3_O_5_, Fig. 1) undergoes a thermal phase transition between a semiconducting (so-called β) and a metallic (so-called λ) phase around *T*_SM_ = 460 K (upon heating)^13,14^. This S-M transition is isostructural^15^ (i.e., it has the same monoclinic space group *C2/m* in both phases, Fig. 1a), is markedly first order and is characterized by significant changes in volume (+6.4%) and latent heat (230 kJ L^−1^)^16^. The increase in volume is mainly caused by expansion of the lattice along the ***c*** crystalline axis. As shown in Fig. 1 the S-M phase transition involves a large intra-cell structural reorganization and the dissociation of the *Ti*_*3*_–*Ti*_*3*_ dimers sharing electrons on a band just below the Fermi level in the β-phase (Fig. 1a, b). From the electronic standpoint, the S-M phase transition is therefore characterized by a vanishing electronic gap related to the dissociation of the *Ti*_*3*_–*Ti*_*3*_ dimers^14,17^. A striking feature of this transition is that the metastability region of the λ-phase depends on the crystallite size. For nano-sized crystallites, the λ-phase is stable down to room temperature (RT) and below, effectively making the system bistable in a broad temperature range^14^. The stabilization of λ-phase is also promoted by doping^18–20^. In the present study, we use a Ti_3_O_5_ pellet of nanocrystals with 72.5% β phase and 27.5% residual metastable λ phase at RT (weight percentages, see Methods). The Ti_3_O_5_ nanocrystals have a typical size of about 100 nm and a “neat-flakes form” morphology as described in^14^. The crystal size was estimated from static synchrotron powder diffraction measurements performed at ESRF ID28 beamline, and described in Fig. S10. X-Ray diffraction (XRD) patterns were recorded for this pellet from 300 to 700 K. Figure 1 panels g and h show the temperature dependence of the ***c***-axis parameter and the monoclinic angle *ϕ* of the different phases. As expected, the λ and β phases coexist from RT to *T*_SM_. The transition temperature of 460 ± 10 K upon heating was determined from these XRD measurements, and it is consistent with the values previously reported for other forms^13,14,16,21^. At higher temperature (T_c_ = 500 K) a second order phase transition occurs towards a high symmetry metallic phase (so-called α, of orthorhombic space group *Cmcm*)^13^. This phase transition is ferroelastic: the change of crystal system from orthorhombic to monoclinic is characterized by a spontaneous shear strain. Thus the monoclinic angle *ϕ* of the λ-phase locks to 90° at T_c_. In contrast to the S-M transition, it is continuous and does not exhibit any volume discontinuity (see orange curve in Fig. 1h). The metastable λ-phase can be switched to the β- phase by applying external pressure (P_MS_ = 0.5 GPa here, but generally strongly dependent on the morphology^16,21^). Also a photo-reversible persistent phase transition between β and λ phases was reported under intense ns irradiation^14,22,23^. Such a photoresponsive phase change material with bistability at RT is of great interest for technological applications, such as optical and heat storage^16,21^. The photoinduced β-to-λ transition was also investigated in the transient regime, below the excitation threshold for permanent switching^22,24^. The full scale dynamics, from fs to µs, was probed by ultrafast diffuse reflection spectroscopy on a nanogranular pellet of Ti_3_O_5_; those measurements were interpreted in terms of nucleation and growth process of λ regions^22^. Unfortunately the previous studies lacked either the time resolution^24^ or the structural sensitivity^22^ to observe strain wave propagation and its role in the phase transition.

## Results

### Time-resolved XRD

**Fig. 2:**
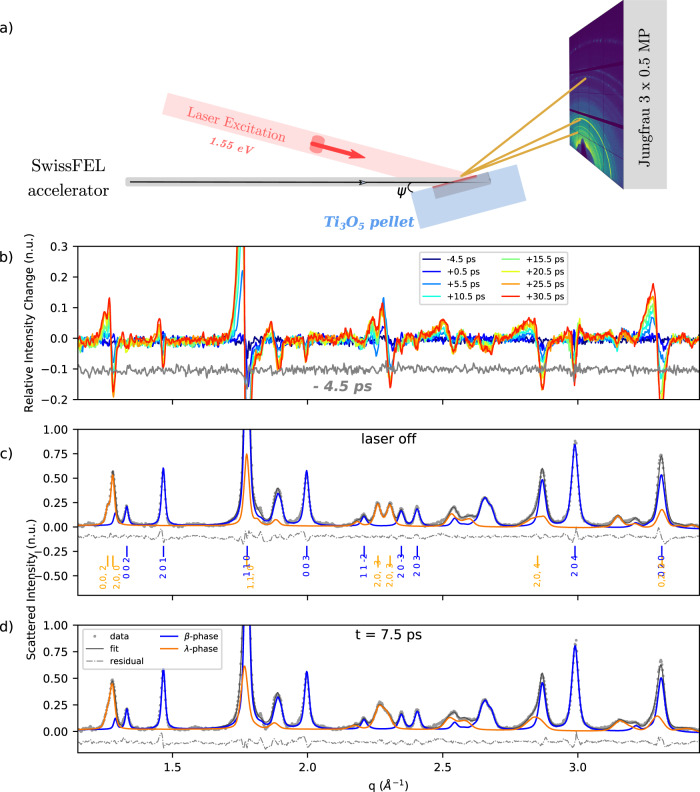
Experimental setup, raw data, and structural refinement. **a** Experimental setup for time resolved powder X-ray diffraction in quasi grazing angle geometry. The Debye-Scherrer rings are collected on a 2D Jungfrau detector with single photon sensitivity^37^. **b** Difference patterns *(laser*_*on*_*-laser*_*off*_*)* showing up to 30% variations of the signal (pump fluence 0.85 mJ mm^2^) on the ps time scale (<35 ps). Gray curve shows the negative −4.5 ps for the non-excited delay for reference (shifted by −0.1 along y for clarity). **c**, **d** Rietveld refinement of reference pattern (pattern with laser off, and pattern at *t* = 7.5 ps respectively). Measured powder patterns are plotted in light gray circles and result of Rietveld refinement in black, solid line. Orange and blue patterns are contributions to the refinement of the λ- and β- phases respectively; the residual curve is shown in gray, dashed lines.

We used ultrafast XRD at the SwissFEL beamline Bernina^25^ to investigate the mechanism of the photoinduced semiconductor (β- phase) to metal (λ- or α- phase) transition in a pellet of Ti_3_O_5_ nanocrystals. The experimental geometry is sketched in Fig. 2a. The X-ray photon energy was 6.6 keV and the grazing angle 0.5°. Considering the X-ray absorption and pellet roughness, the effective probed depth for this geometry is *z*_*p*_ = 400 nm (Figs. S2 and S3). The 1.55 eV, 500 fs long pump pulse impinges on the sample at an incident angle of 10°. At this energy, the pump penetration depth calculated from the refractive index is ξ_L_ = 65 nm^26^. The measured time resolution is about 600 fs FWHM^25^. These studies are complemented by experiments at the ID09 beamline at ESRF, probing longer time scales with a time resolution of 100 ps (Fig. S4). Unless stated otherwise, we will refer to the SwissFEL experiment in the following. Figure 2b compares difference powder patterns taken at various time delays. The low noise, featureless transient at −4.5 ps, shows the high quality of the data and data reduction procedures. The difference curves in Fig. 2b show that major changes are already present at 500 fs which indicates rapid structural deformations. On the picosecond time scale the difference signal increases in amplitude; the signal around some Bragg peaks, for instance (110) and (020), changes towards a characteristic “bi-polar shape”, i.e. positive change towards low angles and negative change towards higher angles indicating an increase of unit-cell parameters. Broadening of some Bragg peaks is also observed (“butterfly shape”), indicating inhomogeneous lattice distortions. Such behavior is clearly observed for the isolated Bragg peak (204) from β phase. The changes in the diffracted intensity suggest that inter-atomic re-organizations take place. The high relative intensity changes, from 10 to 30% depending on the *q* region, indicate that large photo-induced deformations/transformations take place in the sample. Despite the complexity of the structural changes, the high-quality data allow to perform a complete Rietveld analysis for all time delays. Figure 2c–d shows the results of the Rietveld refinement of the diffraction patterns for a reference pattern (laser OFF) and for the 7.5 ps time delay (see also Fig. S5). The full *q* range measured and used in the refinement is displayed, namely 1.09–3.45 Å^−1^. The Rietveld analysis for equilibrium and time resolved data, with unconstrained cell and atomic position parameters for the λ- and β-phases, have similar reliability factors (*R*_p_ = 3% and *R*_wp_ = 7–8%). This attests the quality of the Rietveld picosecond analysis which motivates examining the evolution of the structural parameters discussed hereafter. All details about the sample, experiment, data reduction, and analysis are given in the Methods section.

### Sub-ps dynamics: structural changes induced by direct optical pumping

**Fig. 3:**
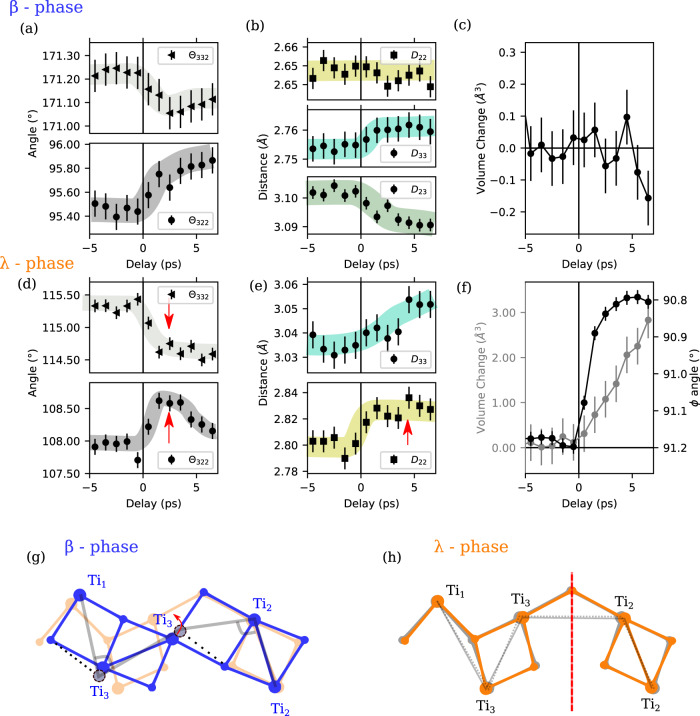
Ultrafast structural changes inferred by X-ray diffraction. Evolution of important inter-atomic angles, distances, and unit cell volume changes for the β- (**a**–**c**) and λ- (**d**–**f**) phases (*ϕ*_*ijk*_ = Ti_i_-Ti_j_-Ti_k_ angles and *D*_*ij*_ = Ti_i_-Ti_j_ distances). The evolution of the monoclinic *ϕ* angle is also reported for the λ- phase (**f**). Thick transparent solid colored lines are guides for the eye. Schematic representation of the observed local distortions (exaggerated for clarity) for the β- (**g**) and λ- (**h**) phases. In (**g**), the λ- structure is represented in light orange for reference and in (**h**), the red line is the *m* mirror planes lost at *T*_c_. A topo of structural distortion shown in gray contour lines: dotted—before, solid—after excitation.

We first describe the ultra-fast structural changes occurring within the first 500 fs where significant intra-cell distortions are already observed. The evolution of selected distances and angles are shown in Fig. 3 for delays up to 7 ps. The typical changes are of the order of 10^−2^ ± 2 × 10^−3^ Å and 0.5–1 ± 0.02°. Comparison with the calculated electronic structure (Fig. 1a–f) helps to rationalize the observed photoinduced changes. The calculated optical conductivity is particularly instructive for describing the possible electronic transitions from the 1.55 eV pump photons (Fig. 1e, f).

For the majority β-phase, the two bands closest to and below the Fermi level are centered at 1.1 and 0.3 eV. They have a strong Ti_2_ and Ti_3_ character, respectively. Although the Ti_2_-Ti_2_ and Ti_3_-Ti_3_ dimers are both excitable above the gap by 1.55 eV photons, the calculated optical conductivity is mainly arising from excitation from the Ti_3_-Ti_3_ dimers to the conduction band. The depletion of electrons in the bonding Ti_3_-Ti_3_ states results in a fast increase of the associated D_33_ distance as observed (Fig. 3b); *D*_ij_ is the distance between the Ti_i_ and Ti_j_ atoms. The dimers also undergo rotation as seen by the decrease in the *ϴ*_332_ angle and increase in the *ϴ*_322_ (Fig. 3a, g); *ϴ*_ijk_ is the angle formed between Ti_i_, Ti_j_, and Ti_k_ atoms. These small amplitude motions are precursor structural signatures of the isostructural phase transition from the β- to λ- phase. However, the rotation is smaller than for the thermal transition and thus results in a significant decrease of the D_23_ distance (contrary to the expected lengthening observed upon thermal transition to λ). On the same time scale, the bond length of the second dimer D_22_ is essentially constant (Fig. 3b), consistently with the fact that D_22_ remains essentially constant in the β- to λ- phase transition.

For the minority λ-phase, the photoexcitation promotes Ti_2_ and Ti_3_ electrons above the Fermi level. The contribution to the calculated optical conductivity from the Ti_2_ and Ti_3_ electrons around 1.55 eV is comparable (see Fig. 1h), albeit they lead to different structural effects. We observe a significant increase in D_22_ at the sub-ps time scale (Fig. 3e): depletion of the Ti_2_-Ti_2_ bonding orbital weakens the Ti_2_-Ti_2_ dimer, and as a result, D_22_ and the non-dimerized D_33_ become less distinguishable. In the high symmetry α phase, Ti_2_ and Ti_3_ are equivalent through a mirror plane (Fig. 3h). The relative evolution of D_22_ and D_33_ (Fig. 3e), as well as *ϴ*_322_ and *ϴ*_332_ (Fig. 3d), directly probes the ultrafast evolution of the degree of symmetry breaking in the monoclinic metallic phase. This points to a distortion towards α atomic structure.

These structural precursors appear before long-range cell deformations are observed (Fig. 3c, f). The increase of the λ- phase volume is linear as a function of time with no discontinuity at time zero; this observation had important consequences discussed in the next section. The rapid decrease in monoclinic angle (plotted in Fig. 3f) indicates that the λ- phase undergoes ultrafast shear with partial symmetry change within 4 ps, slower than the sub-picosecond structural changes but faster than the change in unit cell volume.

### Dynamics of long-range structural changes

The dynamics of the aforementioned long-range cell deformations will now be addressed. First the results of the Rietveld refinement will be presented followed by the discussion on a phenomenological model to explain the data.

**Fig. 4:**
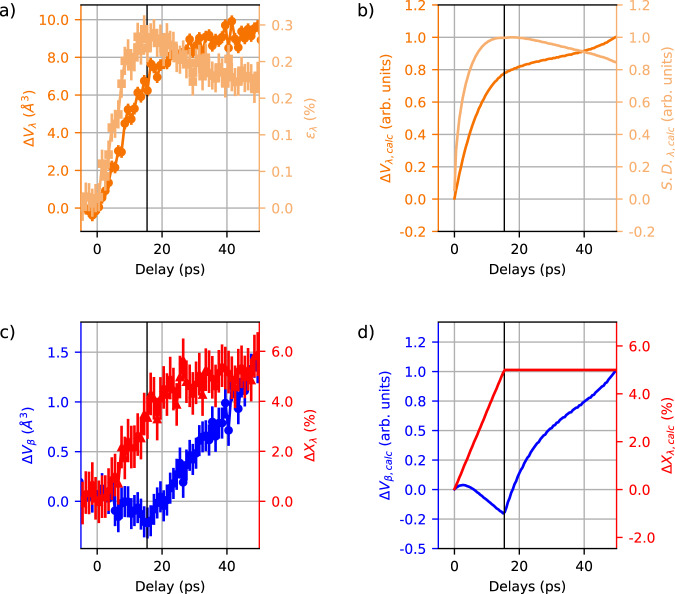
Evolution of long range order and phase change on the acoustic time scales. **a**, **c** Parameters extracted from Rietveld refinement of the XRD data. **a** Temporal evolution of the λ- phase unit cell volume (Δ*V*_λ_, dark orange, filled circles) and microstrain parameter *ϕ*_λ_ (light orange, filled squares). **c** Temporal evolution of the β- phase unit cell volume (Δ*V*_β,_blue) and λ- phase fraction (Δ*X*_λ,_ red). Relative changes normalized to the change at 50 ps and errors estimated as described in the Methods section. **b**, **d** Simulation with the Thomsen model^11^ of the parameters shown in (**a**, **c**). All details are given in the SI. **b** λ- crystallites. Calculation of the expected evolution of the volume change (Δ*V*_λ, calc,_ dark orange) and microstrain (S. D._λ, calc,_ light orange) following ultrafast excitation. Parameters of the model are taken as follows: laser penetration depth of *χ*_L_ = 65 nm, sound velocity of *v*_s_ = 6.5 × 10^3^ m s^−1^; curves normalized to the value at *t* = 50 ps. **d** β-crystallites. Calculation of the expected evolution of the volume change (Δ*V*_β,calc_, blue) and λ- phase fraction (Δ*X*_λ,calc,_ red) following ultrafast excitation. The corresponding acoustic front propagation time corresponding to *z*_S_ = 100 nm, *ϕ*_PF_ = *z*_S_/*v*_s_) is shown with solid black lines in (**a**, **b**, **c**, **d**).

The evolution of the λ- phase unit cell volume takes place on the 10 ps time scale (Δ*V*_λ_, Fig. 4a). The increase is linear up to *t* ≈ 16 ps; In this time range a strong broadening of the Bragg peaks is also observed. It results from the strain distribution, described here by the microstrain parameter (*ϕ*_λ_) as conventionally used in the analysis of powder diffraction. The evolution of the λ- macroscopic phase fraction (Δ*X*_λ_, Fig. 4c), signals that part of the β- crystallites undergoes the phase transition. At 20 ps, *X*_λ_ increases from 27.5% to 33.0 ± 0.8% on average within the X-ray probed depth (400 nm). Importantly its evolution is also quasi linear up to *t* ≈ 16 ps, thereby the dynamics of the λ- phase fraction follows the evolution of the λ- phase unit cell volume. This suggests that the underlying mechanism is governed by the propagation of elastic deformations contrary to the often purported “nucleation and growth”. In the latter case, the transformation can be described as a sequence of steps: after an initial formation of the seed, a relatively fast growth of the nuclei follows until the nuclei encounter boundaries that slow the process again. For this reason, such models often result in temporally sigmoidal increase of phase fractions^27^, which remains in stark contrast with our observation on Ti_3_O_5_. The experimental data suggest that the metallic λ phase is created coherently within a traveling deformation. This motivated us to develop a strain-wave model discussed below.

The behavior of the β-phase unit cell volume is more complex. The Δ*V*_β_(t) curve (Fig. 4c) has a minimum around *t* ≈ 16 ps whereafter the volume increases to the equilibrium value and even above. The β- volume increases from 349.3 to 350.7 Å^3^ (+0.4%) which is rather small compared to the increase from 371.7 to 381.3 ± 0.1 Å^3^ (+2.5%) in the metallic phase.

### Strain-wave model

The connection between the ultrafast photo-excitation and the strain dynamics leading to the overall volume change is described by Thomsen^11^ and this model has been used successfully to describe strain propagation^12,28^. We performed numerical calculations for the λ- and β- crystallites separately, in order to rationalize our observations (see SI “Model calculations of Strain, Volume change and Microstrain”). In the case of a metal, the original model assumes that stress is set up by lattice heating. In the case of semiconductors, the creation of electron–hole pairs results in a more complex contribution, separated by Thomsen as electronic (proportional to deformation potential^29^) and phononic (thermal phonon, proportional to excess energy *E*_ph_–*E*_g_, respectively photon and gap-energy). In the present case however, the stress arises primarily from the precursor photoinduced electronic changes and the structural distortions described in the previous paragraphs, which we argue will lead to phase transition. We consider the same semi-infinite medium as in the seminal work by Thomsen^11^. We also assume that the initial stress profile decreases exponentially due to the absorption of the laser pulse. To establish the mechanical equilibrium between crystallites and their environment, acoustic wave packets propagate from the surface inwards, leaving behind a static deformation. Note that electronic diffusion, and how it affects the strain wave, is neglected in the model. Although this process probably occurs, it is not needed for reproducing the experimental data. The strain wave is described as follows^11^:

$${\eta \left( {z,t} \right) = S \times f\left( {z,t} \right)}$$

where $${f\left( {z,t} \right) = \left[ {e^{ - z/\xi }\left( {{\mathrm{1}} - \frac{{\mathrm{1}}}{{\mathrm{2}}}e^{ - v_st/\xi }} \right) - \frac{{\mathrm{1}}}{{\mathrm{2}}}e^{ - \left| {z - v_st} \right|/\xi } \times sign\left( {z - v_st} \right)} \right]}$$,

S is the strain at *z* = 0, where the stabilized deformation is maximal. It determines the amplitude (and sign) of the volume change induced by the initial stress. The time dependence *f(z,t)* determines the profile of the acoustic pulse, travelling at the speed of sound *v*_*s*_, as well as the static deformation left behind. The speed of sound, 6.5 × 10^3^ m s^−1^, was determined independently with picosecond interferometry^30^, sensitive to propagating acoustic wavepackets^22^ (Fig. S8). In the following, the time *t* and position *z* are related through *v*_*s*_.

Based on this approach we performed numerical calculations, including the different possible responses of the two phases. The calculations qualitatively reproduce the experimental changes in lattice volume, microstrain and phase fraction as shown in Fig. 4.

**Fig. 5:**
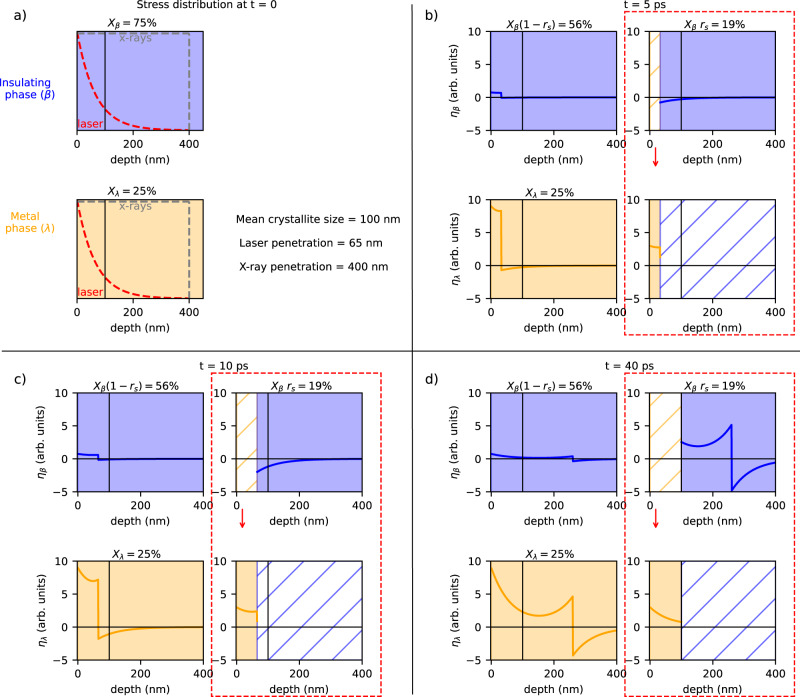
Strain wave model with phase transition. The spatial dependence of the elastic strain at different times (tagged top of each panel), for β-phase crystalites (in blue, initial fraction *X*_β_ = 75%) remaining in the initial phase (56%, left side on each panel) and transitioning to λ (19%, right side on each panel), λ-phase crystallites (in orange) remaining in the initial phase (initial fraction *X*_λ_ = 25%, left side on each panel) and newly formed from β (19% with transformation rate *r*_s_ = 0.26, right side on each panel). To distinguish the strained-transitioning crystalites from those strained-non transitioning, the former situation is contoured on each panel with a red dashed line and red arrow points to the transition direction. The solid vertical line at z = 100 nm on all panels delimits the phase-transition zone. Semi-infinite 1D medium approximates the real sample and the experimental conditions. **a** Initial photo-induced stress following the laser absorption profile (in red). X-ray probe depth is indicated in gray (details given in Fig. S9). The inset shows the most important values in the experiment. **b**–**d** Strain profile η(*z*,*t*) as calculated according to the model described in the main text. For transitioning crystallites (contoured dashed red) the strain due to the transition is calculated separately for the λ- and β-phase.

For the β crystallites, (75% of sample before excitation) we first consider the case where no phase transition occurs (56% of the sample). Then, the maximum volume expansion has an amplitude *S*_β, T_ ∝ *V*_β_(*T*_c_) − *V*_β_ (*T* = 300 K) = 1.5 Å^3^. The time evolution is strictly that proposed by Thomsen (Fig. 5b–d, upper left panel). The presence of a phase transition (19% of the sample) increases the complexity of the model. First, the expanded region is assumed to transition to the λ phase, and as such it no longer contributes to the strain in the β phase (Fig. 5b–d, right panels). The associated stress leads to an increase in volume expressed as *S*_β, PT_ ∝ *V*_λ_ (*T* = 300 K) − *V*_β_ (*T* = 300 K) = 20 Å^3^ (see Fig. S7), and a significant compressive wave is launched (Fig. 5b, c). Hence the second consequence: the volume shrinkage of the β phase near the phase front, as observed experimentally, see Fig. 4d. The transformation stops at (*t* = τ_PF_, *z* = *z*_s_), yet the strain wave generated at *t* = *0* continues to travel into the region *z* > *z*_s_ of the β phase and induces a positive strain therein.

Even though we cannot distinguish experimentally between transitioning and non-transitioning β crystallites, the respective contributions *S*_β,PT_, and *S*_β,T_ determine the sign and amplitude of the average negative change of volume observed at τ_PF_. The amplitude of *S*_β, PT_ is ~13 times greater than that of *S*_β,T_ as shown above, in agreement with the observation (Fig. 4c, d). However, any ratio in the range^10–20^ would still give reasonable agreement.

For the λ crystallites, we first consider the lattice heating from the photo-excitation of the λ crystallite (25% of sample before excitation). For the calculation of the average strain in this phase, we also have to include the new λ crystallites transitioned from β. The two contributions *S*_λ,PT_ and *S*_λ,T_ are both of phononic origin and scale with the energy deposited in the lattice. For the new λ phase, the energy transferred to the phonon bath is equal or less than *E*_*ph*_ *−* *E*_*g*_ *−* *Q* = 0.55 eV (*E*_g_ = 0.7 eV, latent heat *Q* = 230 kJ L^−1^ as reported in^16^, corresponding to 0.25 eV/dimers(Ti_3_-Ti_3_)). In the excited pre-existing λ phase, the full photon energy (1.55 eV) is assumed to be transferred to the phonon bath. The calculations presented in Figs. 4 and 5 are performed with the amplitude of *S*_λ,PT_ = 1/3 *S*_λ,T_, however, the variation of the relative contributions in the range [0–0.5] would still give a reasonable agreement.

It is important to explicitly mention that in order to limit the number of adjustable parameters and keep the discussion of the model as simple as possible, we have made the following assumptions:

- Nanocrystals are considered somewhat independently from one to another except along the depth direction.
- Each nanocrystal is assumed to be monophasic before photoexcitation
- Each nanocrystal is assumed to be either fully transformed after the transit of the strain wave or not transformed at all.

These hypotheses seem reasonable based on our present knowledge. In the strain model, this choice was made for the sake of simplicity as they allow to describe the entire sample as “weighted sum” of three contributions that are described below in details: λ nanocrystals for which excitation translate into lattice heating, β nanocrystals that transform into λ and β nanocrystals that remain in the β phase and are simply heated up by the photon absorption.

An alternative hypothesis would consider a mixture of β/λ domains in the crystallites before and after transformation. In this case, one would need to consider an extra compression due to the transition on the untransformed domains. Calculations including this extra contribution are displayed in Fig. S11, showing no major difference with the evolutions displayed in Fig. 4b, d.

Details of the calculations of the parameters shown in Fig. 4b, d are given in SI. The strain gradient, arising from the initial stress distribution, is quantified by the standard deviation of the calculated strain (Fig. 4b). It is then compared with the observed Bragg peak broadening, quantified in the Rietveld refinement by the microstrain parameter ε_λ_ (Fig. 4a).

## Discussion

This simple model is an attempt to generalize the Thomsen model to include a phase transformation. This is essential for systems where the volume change associated with the transition is comparable (or higher) that the laser induced thermal expansion included in the original model. For the β-phase, we accounted for a partial transformation of the crystallites, and the associated change in the unit cell volume. The initially excited β-crystallites are assumed to be promptly transformed to λ- phase concomitant with the moving acoustic wavefront. This model has its limits, most notably the feedback mechanism between volume change and transition is not rigorously solved by the model. In spite of that, the physical picture of a phase front moving with the acoustic front agrees well with the experimental data. The phase front is assumed to stop at *z*_s_ = 100 nm, matching the average crystallite size as measured by XRD. The acoustic front takes *τ*_PF_ ~16 ps to travel this distance (see the black line in Fig. 4a–d). This is exactly when the β-phase volume shows a well defined minimum, a direct consequence of the compression exerted by the high volume of the layer that transforms to the λ-phase (Fig. 5). The latter also influences the calculated mean strain in the λ-phase (Fig. 4b). The λ-phase fraction is plotted in Fig. 4d in agreement with the behavior in Fig. 4c. As a result, the 5% average increase within the X-ray probed depth of *z*_p_ = 400 nm, amounts to ~26% within *z*_s_ = 100 nm, where the phase front stops (see Fig. S9). Since the laser penetration, average crystallite size, and the phase-front breakdown limit are all about 100 nm, we cannot speculate on the exact nature of the transition limit. Only a quarter of the crystallites undergoes transition by the strain wave, which we tentatively assign to residual static stresses pre-existing in the sample (λ/β coexistence) and/or to the random orientation of crystallites. The latter would be related to the predominant role of expansion along the *c*-axis to stabilise the new phase. Finally, the exact origin of the phase transition requires further information about the nature of the λ-β interface. Our study probes the bulk of volume penetrated by the X-rays, so we cannot claim that the transition at the interface is driven by the strain alone. Yet, the strain and the phase transition propagate at the same velocity showing that the strain wave determines the pathway of a truly macroscopic semiconductor to metal transition in nano materials that exhibit a volume change during the phase transition. Relying on the same physical picture, the 4 ps shear dynamics described above, would correspond to propagation on 10 nm with a transverse acoustic velocity of 3 × 10^3^ m s^−1^^16^. High resolution single crystal studies of the same compound performed at ID28 (ESRF), suggest the presence of relatively dense stacking faults along the *c* axis (Fig. S6). Ferroelastic domains of similar size were also recently reported for an archetypal oxide^31^.

**Fig. 6:**
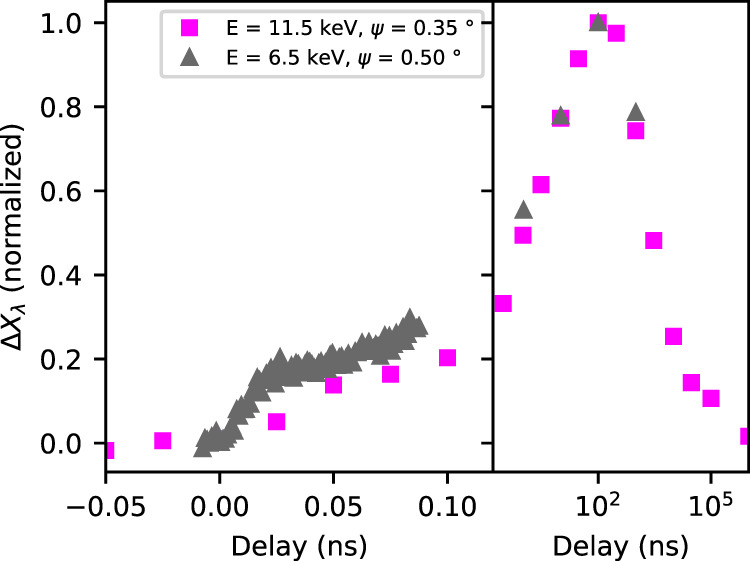
Thermal propagation in the bulk pellet. Multiscale evolution (from ps to ms) of the normalized λ fraction (Δ*X*_λ_) as extracted from time resolved XRD measurements at the ESRF beamline ID09 (magenta squares, X-ray energy *E* = 11.5 keV, incident angle ψ = 0.35°). The fractional change is normalized to the maximum value for allowing a direct comparison with the evolution observed at SwissFEL Bernina (gray triangles). Long time delays are presented on a logarithmic time scale.

On longer time-scales (*t* > τ_PF_), the temperature and strain distributions persist until heat diffusion equilibrates the temperature over the probed area^11,12^. This was investigated by XRD with 100 ps time resolution at the beamline ID09 at ESRF^32^ (Fig. 6). The slow process can be clearly distinguished because it develops until 100 ns. The fraction of transformed β- crystallites reaches 30% in the probed volume (see Fig. S9). The 100 ns value is consistent with expected time for heat diffusion over *D* ≈ 200 nm (in a bulk material with thermal diffusivity of 230 nm^2^ ns^−1^)^16^. The complete recovery of thermal equilibrium with the environment is observed on a 10 µs time scale, in agreement with^24^.

This work offers a new perspective for ultrafast control of materials with evidence of a strain wave pathway for achieving a phase transition in nanocrystallites excited by a light pulse. The key role of the long-range lattice deformation is highlighted, and so are the benefits of direct structural probes for visualising thereof. We demonstrate that state-of-the-art XFEL sources permit the probing of the interatomic motions and lattice distortions in real time, even in a bi-phasic polycrystalline powder. Our experiment, corroborated with a phenomenological elastic model, reveals unambiguously that a phase front starting from the surface is moving with sonic speed into the bulk. This process, restoring mechanical equilibrium, takes place on an ultrafast timescale and clearly precedes the much slower thermal diffusion homogenization. XFEL data suggests that the transition propagates coherently, as opposed to random growth induced by incoherent thermal kinetics (observed with synchrotron pulses on 100 ns time scale). Indeed nucleation-and-growth models often result in a sigmoidal temporal dependence of the new-phase fraction^27^, which is markedly different from the growth we observed. The simultaneous change of the λ-phase fraction and its volume substantiate our interpretation.

We believe that the strain wave pathway is likely to be valid in a variety of other volume changing materials. Notably, the strain waves are launched directly in the material by internal precursor stresses that store mechanical energy. This is a step forward for self-contained transformation, in contrast to previously reported phase front propagation which required a hetero-structures for launching where the strain waves are created at the interface of a strongly absorbing transducer film into the phase change film^33,34^. We show that our approach is nondestructive, and opens up fascinating prospects for an advanced degree of strain control through electronic and structural precursors.

## Methods

### Static powder laboratory XRD—temperature measurements

The static powder XRD as a function of temperature was measured in Debye-Scherrer geometry on a Bruker AXS D8 Advance (Mo-K_α_ radiation selected with a focusing Göbel mirror) equipped with a MRI high temperature capillary furnace and a high-energy LynxEye detector. The flake form Ti_3_O_5_ powder sample^14^ was sealed in a quartz capillary of 0.3 mm in diameter.

### DFT/optical properties calculations

All calculations have been carried out by using the Quantum ESPRESSO package within the framework of DFT + U, with the Perdew-Burke-Ernzerhoff generalized gradient approximation to describe the exchange-correlation functional. Both Projector Augmented Wave basis and norm-conserving pseudo-potentials. The Monkhorst-Pack grid of 8 × 8 × 4 in the reciprocal space was used for the Brillouin zone sampling for both λ- phase and β- phase. The total energy of the system converged to less than 1.0 × 10^−6^ Ry. Electronic wave functions were represented in a plane wave basis up to an energy cut-off of at 90 Ry. Crystallographic structures were taken from^14^. Ferromagnetic and antiferromagnetic (AFM) orders were considered. AFM order was found to be the ground-state for both λ- and β-phases. The optical properties were computed using epsilon.x post-processing tool of the Quantum Espresso package, at the independent-particle approximation level. Both intraband and interband contributions were considered. Calculated optical density shown in Fig. 1 is the average of the 3 diagonal elements (Fig. S1).

### Samples preparation

Flakes-form λ-phase samples were obtained following the synthesis method described in^14^. Pellets were made from flakes powder using a uniaxial press at 3 GPa. The resulting pellets have a density of 3.2 g cm^−3^ (a ratio of 0.8 compared to single crystal, measured by X-ray absorption). They contain a mixture of pressure-induced β-phase crystallites and λ-phase crystallites due to residual stress. The absolute λ-phase fraction is determined by Rietveld refinement around 25–30% (depending on the pellet), in good agreement with the ratio usually observed with this method^14^. Whether β- and λ- could coexist within a single grain remains an open question and cannot be answered with our XRD measurements.

### Experimental details—time-resolved experiment

Experiments were performed at ESRF Synchrotron (Grenoble, France) using the ID09 time resolved beamtime (exp1) and at SwissFEL (PSI, Villigen, Switzerland) at the Bernina beamline (exp2). The latter was part of the very first commissioning experiment of the entire facility.

### EXP1: ESRF experiment detail

The ID09 setup consists of fast rotating choppers to select to isolate X-ray single pulses (each 100 ps long) at 1 kHz repetition rate^32^. A fast shutter was used to lower the frequency to 10 Hz. The X-rays were partially monochromatized by using a Ru/B_4_C multilayer monochromator resulting in 1.5% bandwidth centered at 11.5 keV and focused by a toroidal mirror to a size of 0.1 × 0.06 mm^2^. In order to reduce the X-ray beam footprint on the sample, the last slits (~0.6 m from the sample) were closed vertically to a 0.03 mm gap. A synchronized laser (800 nm) at 10 Hz was used to excite the sample using a perpendicular geometry configuration, with the laser hitting the sample from the top. Laser Beam size was 10 × 0.23 or 4.6 × 0.21 mm^2^ depending on power densities (respectively below and above 1 mJ mm^−2^). Diffracted X-rays were integrated on a Rayonix MX170-HS CCD detector and azimuthally integrated using pyFAI^35^.

### EXP2: SwissFEL experiment

The experimental setup is shown in Fig. 2a. The SwissFEL produces short X-ray pulses (estimated duration 300 fs) that are sent directly to the Bernina beamline^25^. Since the experiment was performed as part of the commissioning for the facility, the X-ray photon energy was limited to 2.2 keV. The third harmonic (6.6 keV) was used to probe the structural changes after photoexcitation. The energy per pulse was ~0.2 mJ and the third harmonic content of about 1%. The fundamental was suppressed using absorbers. We estimate about 10^9^ 3rd harmonic photons per pulse at the sample position. The X-rays were focused by a Kirkpatrick–Baez mirror system. The spot was set to ~200 × 7.5 μm^2^ (FWHM h × v). Because of the small grazing incidence angle of 0.5° the small vertical spot was essential to minimize the footprint on sample which with a length of 0.86 mm footprint is still one of the major contributions to the peak broadening. The pump laser pulses, generated by a Coherent CPA amplifier (Legend) were purposely stretched to 0.5 ps to limit the peak intensity on the sample increasing the overall time resolution to ~600 fs. Note that no jitter correction^36^ was used for the experiment. The measured instrumental time resolution of 350 fs FWHM^25^ increased due to the pump pulse stretching to about 600 fs. The laser beamsize at the sample position was 300 μm (FWHM). With an incident angle of 10° the resulting footprint was 300 × 730 μm^2^. The repetition rate of the laser was set to 5 Hz minimizing residual heating effect.

### Data reduction/correction

Diffracted X-rays were measured (for every single pulse) by the Jungfrau pixel detector^37^. The detector was calibrated to convert ADU directly in equivalent keV photon energy. Most pixels (>70%) had zero counts, the others detected either one elastic photon (~6.6 keV reading, about 3% of the pixels), a Ti fluorescent photon (centered around 4.4 keV, 11% of the pixels) or a combination of the two (1 elastic +1 fluorescence photon). By eliminating the fluorescence photons from each image a lower background image was obtained. Each “fluorescence corrected” image has been azimuthally integrated to obtain 1D curve^35^. Each data point was collected by acquiring 500 images (250 with pump laser and 250 without pump laser). The two sets were averaged (after “fluorescence correction” and azimuthal integration) to provide two curves (laser off and on). To correct for drifts in photon energy each “off image” was used to extract the average photon energy during collection of a given time delay. The corresponding “laser on” image was treated using the optimized X-ray photon energy. To further verify the stability of the data collection and correction strategy, every 10 delays a reference time delay was acquired to be sure that no drifts or permanent sample change had happened. Each powder pattern was normalized to the intensity of the air scattering contribution dominating at low scattering vectors (between 0.7 and 0.9 Å^−1^). All raw data and data reduction scripts will be made available.

### Data analysis (Rietveld refinement)

Rietveld whole powder pattern profile refinements were performed following a fundamental parameter approach using the TOPAS software^38^. Pawley refinements were also used and both methods gave consistent evolutions of the unit cell parameters and scales factors. In both cases, refinement was performed using the *q* range from 1.09 to 3.45 Å^−1^, corresponding in this case to diffraction angles from 19° to 63°. The profiles were described using a beam energy of 6.5 keV (λ = 1.899 Å) and a gaussian emission profile with 1.3% FWHM. The peak width and sample displacement were described using the expressions from^39^, vertical width and incident angle of the beam being fixed as measured (4 µm and 0.5° respectively). The sample detector-distance was fixed to 61 mm, as refined in the azimuthal integration step. The absorption coefficient at 6.5 keV was calculated as 800 cm^−1^ (for a packing density of 0.8, measured from x-ray transmission measurement). These parameters were fixed for the refinements against time resolved patterns. The refinements included two phases (β- and λ-). For each phase, the free parameters were the cell parameters (a, b, c, *ϕ*), the atomic position in the (***a***, ***c***) plane for the five independent atoms (position along ***b*** being symmetry-restricted) and the scale factor. An additional microstrain-type gaussian convolution (FWHM = *ɛ* × tan(*θ*)) accounted for the strain distribution observed during the propagation (Fig. 5). The initial Bragg peak profile was well defined by the experimental resolution function defined as described above. Thus no extra size contribution was considered in the refinement of the reference patterns. The transformation being reversible and isostructural, no evolution of the crystallite size was expected and thus considered either. In any case, the choice was made to retain the simplest convolution functions to ensure the robustness of the refinement. Hence no lorentzian contribution, asymmetry or anisotropy was taken into account. The background was described as a 3rd order polynomial. The texture of the majority phase was described using Spherical Harmonics corrections, whose coefficients were refined on reference patterns and then kept constant for all delays. The *R*_wp_ agreement factors were around 7.5%; *R*_Bragg_ were calculated around 2% for the majority β- phase and around 7% for the λ- phase. For the SwissFEL data refinement, errors were estimated from the distribution (standard deviation *SD*) of the refined values obtained on reference pattern (interleaves data with no laser), and thus have to be understood as relative errors; the errors given in the text and in the figures corresponds to *2*x*SD*. Note that for the ESRF data, the quality of the extracted patterns did not allow to refine the atomic positions. In both cases, reference structures were taken from^13,14^. The atomic displacement parameters could never be refined accurately due to the limited *q* region measured and large beam footprint resulting in poor *q*-resolution. These values were fixed with those reported in the literature.

## Supplementary information

Supplementary Information

## Data Availability

Raw data for the SwissFEL experiment, scripts to process them and to produce the final figures of the main text have been uploaded to the Zenodo repository and can be found here: 10.5281/zenodo.4327604. All other data can be provided upon reasonable request.
