# Supplementary material for: Strain wave pathway to semiconductor-to-metal transition revealed by time-resolved X-ray powder diffraction
Source: Nat Commun. 2021 Feb 23;12:1239. doi: 10.1038/s41467-021-21316-y (PMC7902810; doi:10.1038/s41467-021-21316-y)
Supplement: Supplementary file 1 — Supplementary Information [file 41467_2021_21316_MOESM1_ESM.pdf]

Supplementary Figures

Optical conductivity calculations

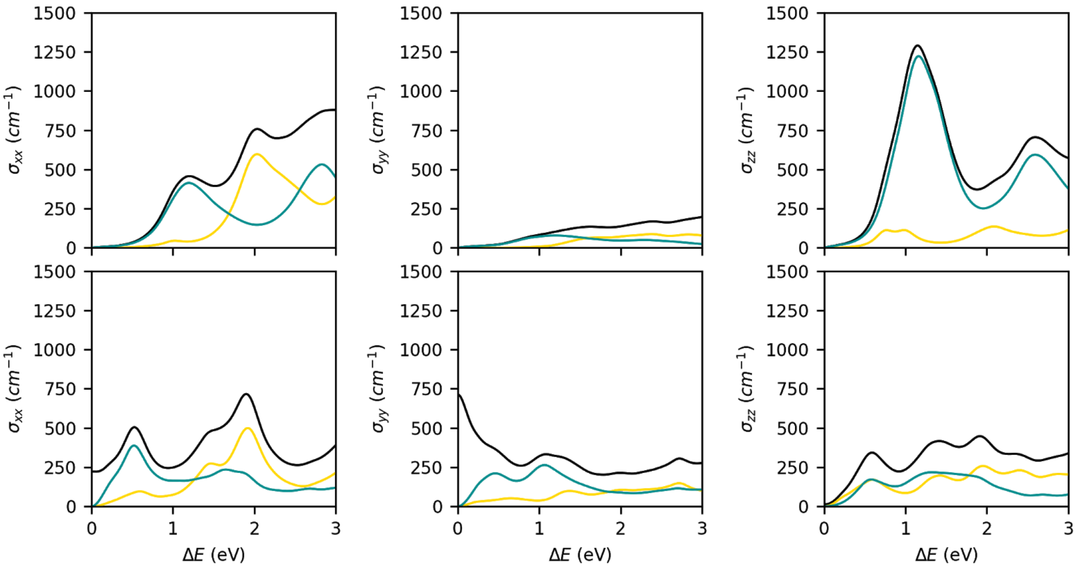

Figure S1: Diagonal contributions of the optical conductivity tensor for the β- (top) and λ- (bottom) phases of Ti_3O_5, calculated along the x, y, z direction of the primitive unit cell. Total calculated conductivity in black; yellow (blue) curve represents the contribution arising from the band at 1.1 eV (0.3 eV) below Fermi level.

Surface Morphology

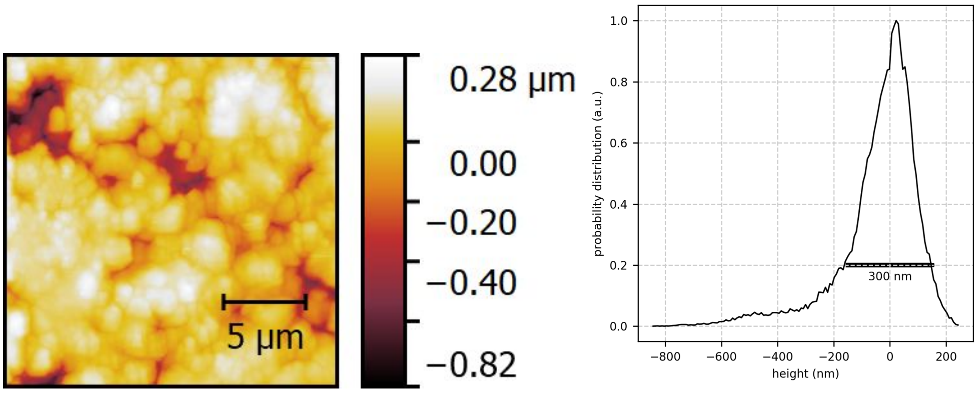

Figure S2 : 20 x 20 µm^2 AFM height image of Ti_3O_5 pellet surface revealing the surface roughness with typical peak to valley amplitude around 1 µm. Such roughness limits the surface sensitivity of the X-ray experiments as discussed in the Methods. Measurement on the very same pellet that was used for the SwissFEL experiment.

Angle dependent penetration depth for a perfect surface

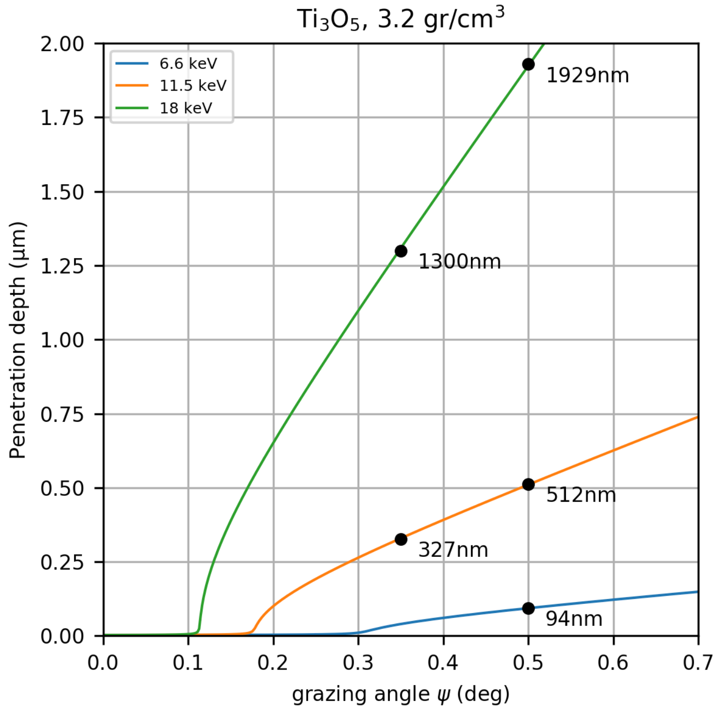

Figure S3: Dependence of the X-ray penetration depth as a function of photon energy and grazing angle, calculated using the [**Henke1993**]; The density for the Ti_3O_5 was set to the one measured by X-ray absorption on the pellets (3.2 g/cm^3). Black circles show the conditions reported in Fig. S4 (right panel).

Long time scale dynamics

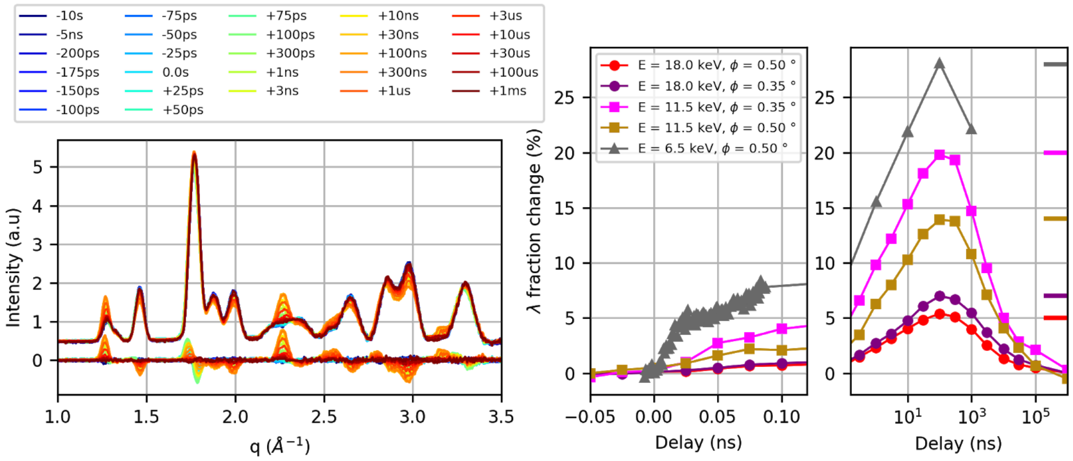

Figure S4: Evolution of the λ- phase fraction from ps to ms time-scales as extracted from TR-XRD data measured on beamline ID09 at ESRF. Right panel) Effect of x-ray probe energy and incidence angle at fixed pump power density (1.84 mJ/mm^2). Data emphasise decreased X-ray penetration into pellets at smaller incidence angles or x-ray photon energy; the reduction of probed depth results into higher λ- phase fraction as described in S9. The Swissfel data are also shown for comparison (grey triangles). The short horizontal lines on the right axis represent the expected fraction of photoinduced λ phase, calculated according to the procedure described in the SI paragraph "Probed Penetration Depth for Ti_3O_5 pellet and switching efficiency"; colours are matched with data. Left panel) Typical time-resolved absolute and differential patterns measured at ID09 (x-ray energy = 11.5 keV, power density = 1.84 mJ/mm^2) The larger beamsize results in longer footprint and reduced q-resolution preventing a full Rietveld refinement of the ESRF data.

Rietveld refinement

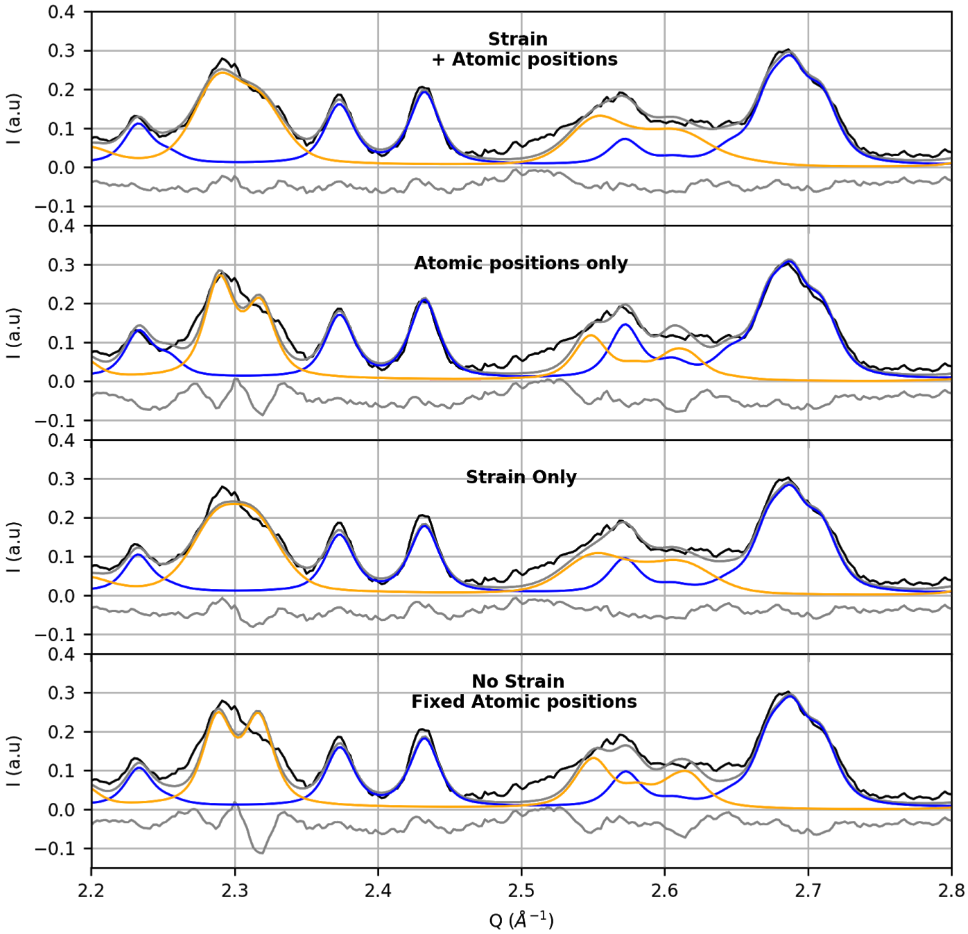

Figure S5: Comparison between different sets of refined parameters, for a diffraction pattern at t = 7.5 ps. In all sets, unit cell parameters a, b, c, ϕ are refined. Experimental patterns are shown in black, refined patterns in grey, contributions of β- and λ - phase in blue and orange respectively, residual values in grey. From bottom to top: atomic positions fixed and microstrain not considered, microstrain refined, only atomic positions refined, atomic positions and microstrain refined.

Diffuse scattering measured of single crystals

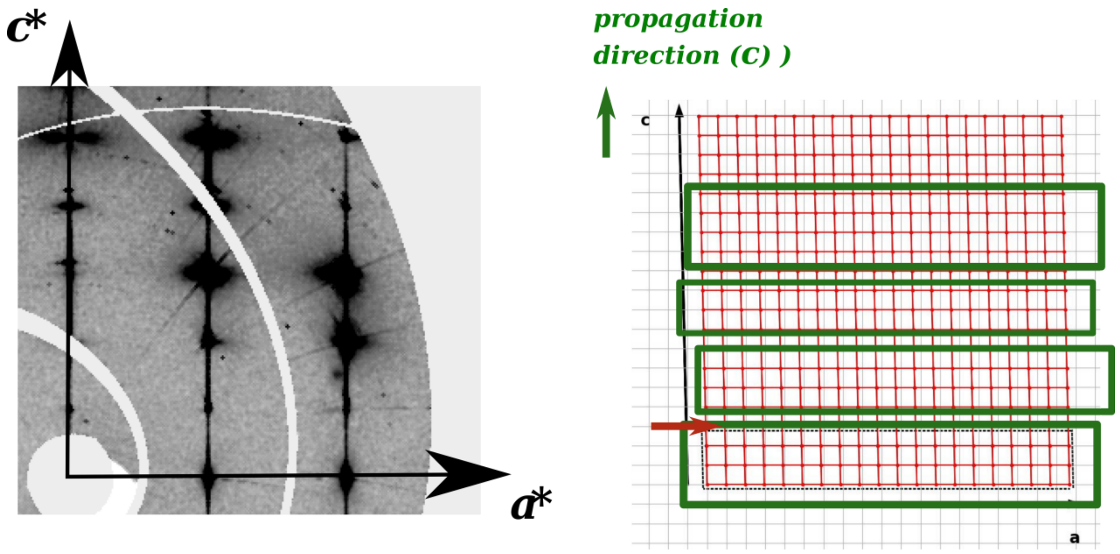

Figure S6: left : (***a****, ***c****) reciprocal plane reconstructed from X-ray diffraction measurements at room temperature on Ti_3O_5 single crystals (360° rotation, 0.1° per images, shutterless mode). Measurement was performed on ID28 beamline at ESRF on a 4-circle diffractometer equipped with pilatus 2M detector. X-ray photon energy was 26 keV. Contrast is enhanced to highlight strong diffuse scattering lines lying along the ***c**** axis, at integer position along ***a****. These lines indicate stacking faults propagating along ***c***, which might arise from the ferroelastic distortion as shown schematically on the right. The exact coherence length could not be quantified precisely, but the intensity suggests only a few unit cells (green rectangle on the right scheme), compared with analogous observations for inorganic crystals [**Zhong2001**].

Temperature dependance of unit cell volumes

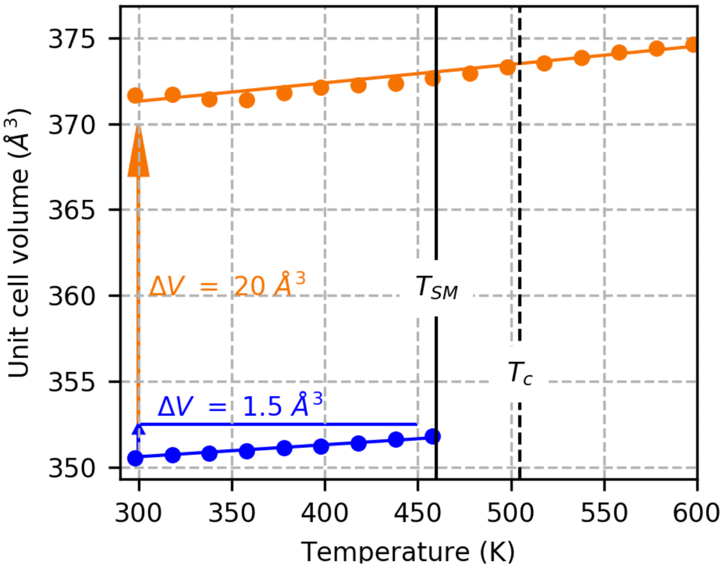

Figure S7: X-ray powder diffraction study on Ti_3O_5 nanocrystals: change in unit cell volume of the β - and λ - phase (respectively in blue and orange) revealing the volume jump of 20 $Å^3$ between the two phase and thermal dilation (1,5 $Å^3$ between T = 300 K and transition temperature T_{SM} = 460 K for β - phase).

Model calculations of Strain, Volume change and Microstrain

In the model proposed by Thomsen and further developed by other authors [Matsuda, Ruello] the mechanical stress has the form:

$$\sigma(z, t) = 3\,\frac{1-\nu}{1+\nu}\,B\eta(z,t) + \sigma_{t=0}(z)\ (eq.\ 1)$$

In the above expression σ represents the stress, ν the Poisson ratio, B the bulk modulus, η the strain, z the distance from the surface. The initial stress $\sigma_{t=0}(z)$ depends on the electron-photon interaction and might include different contributions.

In the case of a metal, the original model assumes that stress is set up by lattice heating and takes the form:

$\sigma_T(z) = -Bb_l\Delta T(z)$ for a "thermal " distribution

where b_l is the linear expansion coefficient.

In the case of semiconductors, the creation of electron-hole results in a more complex contribution, separated by Thomsen as electronic (depending on deformation potential [ref Matsuda]) and phononic (Thermal phonon, depending on excess energy $E_{h\nu}$-E_g). :

$\sigma^e_{ij}(z) = -B\frac{dE_g}{dP}\delta_{ij}\delta n_e(z)$ electron-hole contribution

$\sigma^p_{ij}(z) = -B\frac{3b_l}{C}(E-E_g)\delta_{ij}\delta n_e(z)$ phononic contribution due to "thermal" electron-phonon relaxation within conduction band; notations taken from [***Thomsen1986***].

In our case we assume that the electron-phonon coupling favours dimers rotation that seeds the phase transition, referred to in the text as local precursors. The density of local precursors is however expected to depend on the excitation density ($\delta n_e(z)$). In either case the excitation density (here $\delta n_e(z)$, or $\Delta T(z)$) , and thus the initial stress, will follow the exponential profile of the laser penetration:

$Ae^{-z/\xi}$, where ξ is the laser penetration depth and A is a constant that depends on the origin of the stress.

With these hypothesis, the strain can be written as:

$$\eta_{ph,\,i}(z, t) = S_{ph,\,i} \times f(T)\ \ ,\ ph = \beta,\ \lambda$$

Where $f(T) = \left[e^{-z/\xi}\left(1-\frac{1}{2}e^{-v_s t/\xi}\right) - \frac{1}{2}e^{-|z-v_s t|/\xi} \times sign(z-v_s t)\right]$

The strain and standard deviation are then integrated between $z = 0$ (surface) and z_p =400 nm (estimated penetration depth of the X-rays) (see Fig. 4 b and d in the main text). The following contribution are calculated:

$$\Delta V_{\beta,calc}(t) = \frac{1}{X_\beta(1-r_s)z_p + X_\beta r_s(z_p - z_m(t))}\left[X_\beta(1-r_s)\int_{z=0}^{z_P} S_{\beta,T}f(t,z)dz + X_\beta r_s \int_{z_m(t)}^{z_P} S_{\beta,PT}f(t,z)dz\right]$$

$$\Delta V_{\lambda,calc}(t) = \frac{1}{X_\lambda z_p + X_\beta r_s z_m(t)}\left[X_\lambda \int_{z=0}^{z_P} S_{\lambda,T}f(t,z)dz + X_\beta r_s \int_{z=0}^{z_m(t)} S_{\lambda,PT}f(t,z)dz\right]$$

$$S.D.^{\lambda}(t) = \sqrt{\frac{1}{z_p + (z_p - z_m(t))} \left[X_{\lambda} \int_{z=0}^{z_P} \left(S_{\lambda,T} f(t,z) - \overline{S_{\lambda,T} f(t,z)} \right) dz + X_{\beta} r_s \int_{z_m(t)}^{z_P} \left(S_{\lambda,PT} f(t,z) - \overline{S_{\lambda,PT} f(t,z)} \right) dz \right]}$$

$$\Delta X_{\lambda,\, calc}(t) = X_{\beta} r_s \times z_m(t) / z_p$$

$z_m(t) = v_L t$ if t < τ_{PF}

$z_m(t) = v_L \tau_{PF}$ if t> τ_{PF}

$v_L \tau_{PF}$ = 100 nm

Z_p = 400 nm the penetration of x-ray

$X_{\beta} r_s$ = 19 % transformed beta to lambda percentage at τ_{PF} (see Fig S9), $X_{\beta}(1 - r_s)$= 56% untransformed beta percentage, X_{λ} = 25 % initial lambda percentage

Picosecond Interferometry measurement

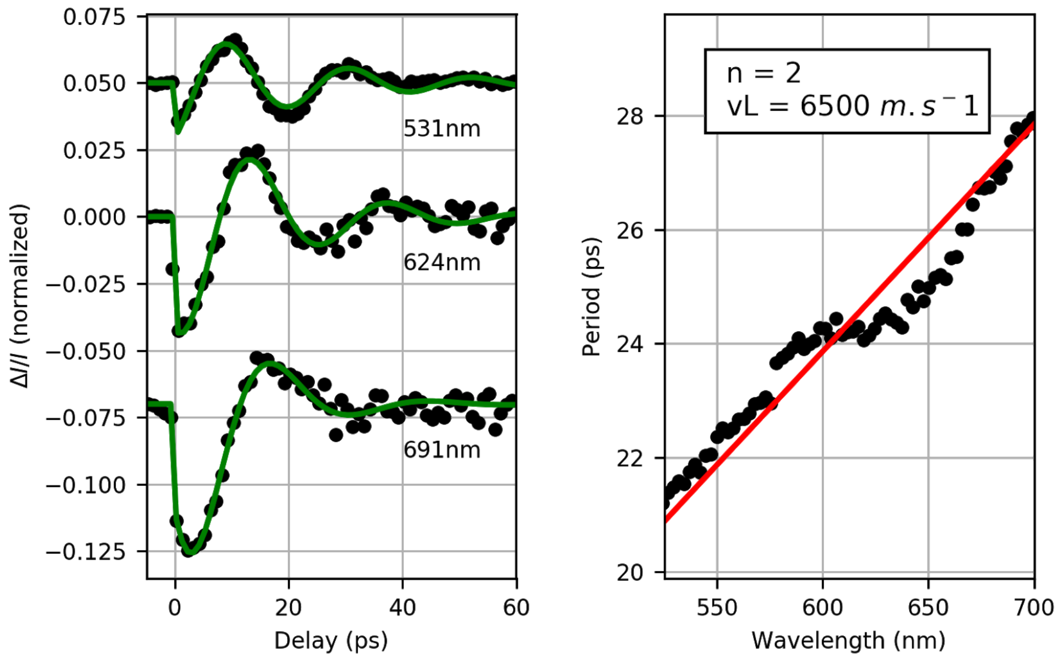

Figure S8: Time resolved reflectivity measured on Ti_3O_5 single crystal with visible pump / white light probe setup at IPR, pump set to 1.55 eV. Left: oscillatory part of the time dependent reflectivity for selected wavelengths of white light probe illustrating oscillation period dependence on wavelength. Right: oscillation period extracted from data refinement. The linear dependence of period was refined following the model proposed in [**Thomsen1986b**]. Extracted periods are in agreement with those previously reported for a pellet sample [**Asahara2014**], and with real part of optical index from [**Hakoe2017**], they yield sound velocity of 6.5e3 $m.s^{-1}$.

Probed Penetration Depth for Ti_3O_5 pellet and switching efficiency

This section aims at obtaining the switching efficiency from the experimentally determined one. The latter one is influenced by the X-ray penetration depth. For ideal surfaces, the X-ray penetration depth can be calculated using tabulated values [**Henke1993**] as implemented using the CXRO website http://henke.lbl.gov/optical_constants/atten2.html. The results for few X-ray photon energies and angles are given in Fig. S3. For sufficiently low angles total external reflection results in an extremely small penetration depth (few nm). The calculated values for the X-ray photon energies and grazing angles shown in Fig. S4 are given in Fig. S3. They range from 92 nm (SwissFEL experiment, 6.6 keV, ψ = 0.5°) to 1.93 µm (ESRF experiment, 18 keV, ψ = 0.5°). The pellet granularity also determines the effective probed depth. The pellets have a typically roughness of 300 nm (Fig. S2). The combined effect of the roughness and ideal penetration depth results in what we call "effective penetration depth" (z_p in the equations below). Since the layer converted by the strain wave (z_s = 100 nm) is smaller than the effective penetration depth (z_p > 300 nm) a smaller apparent phototransformed fraction will be observed.

By using a simplified model shown in Fig. S9 we can calculate the apparent fractions as a function of relevant physical parameters. In particular we assume that strain wave propagation and heat diffusion processes transform a finite fraction of the β phase, and the respective efficiencies are denoted r_s and r_h.

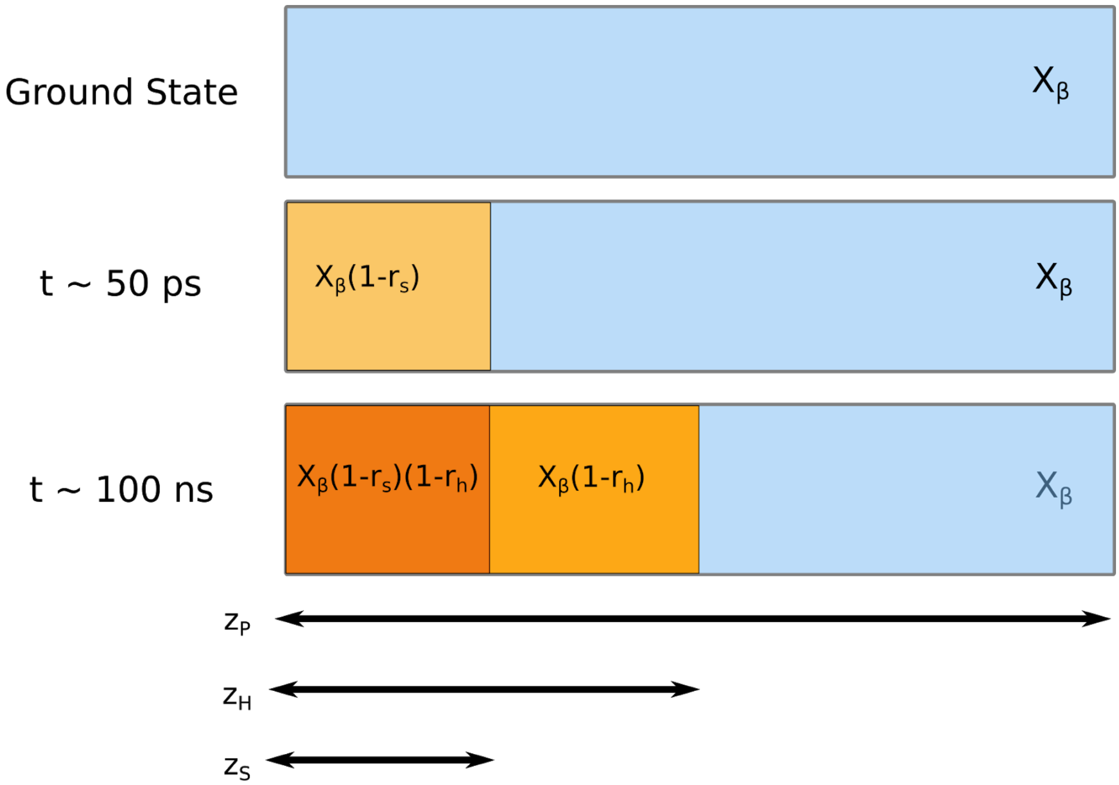

Figure S9: Model used to calculate the switching efficiencies. The horizontal axis represents the sample thickness. Without photoexcitation the sample is in a mixture of λ and β phases indicated respectively as X_λ (~0.25) and $X_{\square}$ (~0.75). After the strain wave propagation a fraction of the β phase will be converted to λ with a certain efficiency (r_s). At longer time extra switching is observed due to thermal transition. In each region the increase of the λ phase is assumed to be equal to the remaining β fraction in that region times the efficiency of "heating

switching" (r_h). Summing the β and λ phase fraction at each step and in each region allows us to calculate the expected measured values.

These models results in simple expressions that link the physical parameters to the observed change of fraction at t ≈50 ps and t≈100 ns:

$$\Delta X_\lambda(50\,ps) = X_\beta r_s \frac{z_S}{z_P}$$

$$\Delta X_\lambda(100\,ns) = X_\beta r_s \frac{z_S}{z_P} + X_\beta(1-r_s) r_h \frac{z_S}{z_P} + X_\beta r_h \frac{z_h - z_s}{z_P}$$

With z_s,z_h,z_p are the strain wave propagation (100 nm), heat diffusion (200 nm) and probed depth respectively. Assuming r_s and r_h to be 0.26 and 0.7, respectively, results in values reported in the table below. The calculated values are obtained from the above expression, the experimental ones from Fig. S4.

	E (keV)	ψ (°)	z_P (nm)	$\Delta X_\lambda(50\,ps)$ (calc)	$\Delta X_\lambda(50\,ps)$ (exp)	$\Delta X_\lambda(100\,ns)$ (calc)	$\Delta X_\lambda(100\,ns)$ (exp)
SwissFEL	6.6	0.5	394	0.048	0.05	0.27	0.28
ESRF	11.5	0.35	627	0.030	0.03	0.17	0.20
	11.5	0.50	812	0.023	0.025	0.13	0.14
	18.0	0.35	1600	0.011	0.01	0.07	0.07
	18.0	0.50	2229	0.008	0.01	0.05	0.05

Size determination

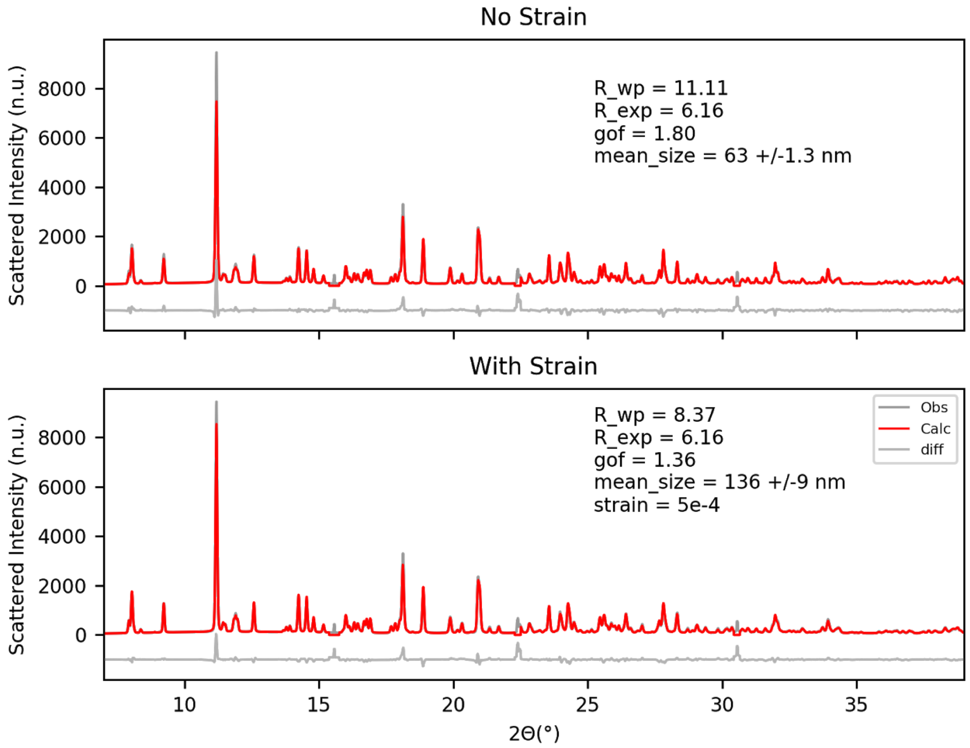

Figure S10: Static powder diffraction patterns used for determination of crystallite size of Ti_3O_5 powder discussed in the main text. Measurements were performed at ESRF, ID28 beamline, using a 2D pilatus 2M detector. The measurements were performed in transmission geometry with a 10 µm diameter capillary. 45 images were recorded in shutterless mode, with 1° rotation step per image. The X-ray energy was 18 keV, and sample - detector distance set to 250 mm. Rietveld refinements were performed using Topas software [***Coelho2018***]. Instrumental broadening was determined based on measurement of Lanthanum hexaboride standart [NIST - SRM660a]. Size and strain broadening were refined using the approach defined in ***Balzar2000*** and ***Bazar2004*** and implemented in Topas [***Coelho2018***]. They were constrained to same value for β- and λ- phase. As described in this article, the size is strongly affected by the consideration of an extra strain broadening. So both cases were considered to estimate the mean size and uncertainty (see upper and lower panels). The mean size is estimated as the average of these two results, namely 100 nm +/- 40 nm.

Balzar2004 D. Balzar, N. Audebrand, M. R. Daymond, A. Fitch, A. Hewat, J. I.Langford, A. Le Bail, D. Loue, O. Masson, C. N. McCowan, N. C. Popa, P. W. Stephens and B. H. Toby, Size–strain line-broadening analysis of the ceria round-robin sample, J. Appl. Cryst. 37, 911–924 (2000) doi 10.1107/S0021889804022551

Balzar2000 D. Balzar, Voigt function model in diffraction-line broadening analysis, in Defect and Microstructure Analysis by Diffraction, ed. by Robert L. Snyder, Jaroslav Fiala, and Hans J. Bunge, Published: 16 March 2000, A International Union of Crystallography Publication, International Union of Crystallography Monographs on Crystallography, ISBN: 9780198501893

Model calculation under different hypothesis

In order to limit the number of adjustable parameters in the strain wave model, the simplest assumptions have been used in the manuscript (independent, monophasic crystals that either transform fully or don't transform at all). Here the result of the model under a different hypothesis is discussed.

Model calculation is done by considering phase mixture and thus extra compression within the first transformed layer (z<100nm) layer. This contribution is scaled by considering the volume jump at the transition (6.4%) and weighted with the percentage of change in the first 100 nm layer (r_s = 0.26, see figure S9). On figure S11, the dotted line plots (without extra compression) have to be compared with the plain lines plots (with extra compression). Importantly we show that considering this extra contribution has no incidence on the features discussed in main text. For the λ phase, the extra compression has a negligible effect on both volume and microstrain evolution as displayed on figure 4. For the β phase, the extra compression of course increases the amplitude of the initial volume decrease. However the shape of the evolution remains the same and the order of magnitude of the minimum remains comparable. We also show the calculation for a slightly different thermal expansion (0.5% for dashed line vs 0.4% for plain line), to underline the relative effect of both contributions..

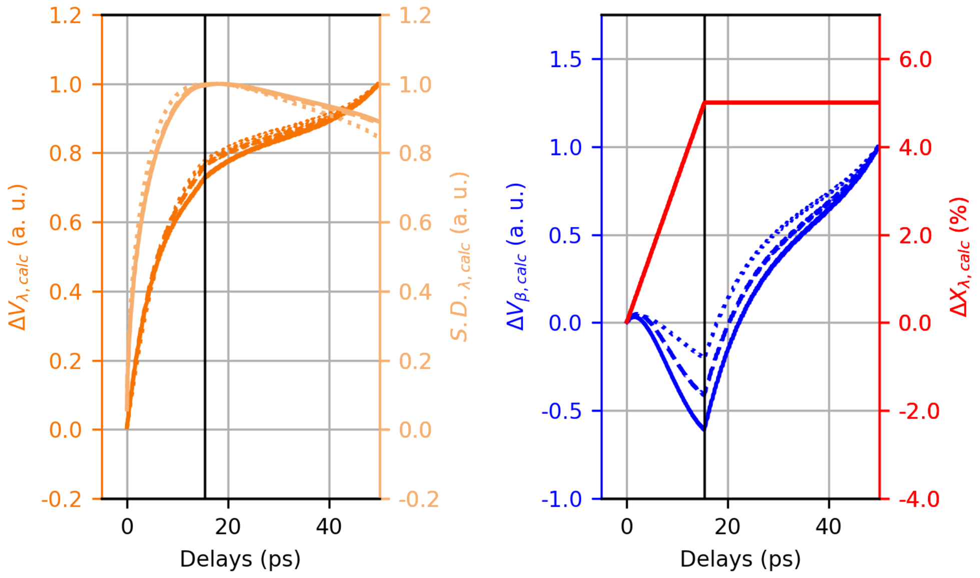

Figure S11: Simulation with Thomsen models of parameters as shown in main text figure 4. Left: volume change and microstrain for λ phase. Right: λ fraction and β phase volume. Dotted lines: simulation similar to figure 4. Plain lines: simulation including an extra compressive strain in the first layer for untransformed region. Dashed lines: same with a higher contribution of the thermal expansion (0.5% vs 0.4%).
